# Outer Membrane Vesicles Transmitting *bla*_NDM-1_ Mediate the Emergence of Carbapenem-Resistant Hypervirulent Klebsiella pneumoniae

**DOI:** 10.1128/aac.01444-22

**Published:** 2023-04-13

**Authors:** Bin Tang, Awen Yang, Peilin Liu, Zhiqian Wang, Zijuan Jian, Xia Chen, Qun Yan, Xianghui Liang, Wenen Liu

**Affiliations:** a Department of Clinical Laboratory, Xiangya Hospital, Central South University, Changsha, Hunan Province, People’s Republic of China

**Keywords:** outer membrane vesicles, carbapenem-resistant *Klebsiella pneumoniae*, horizontal gene transfer, *bla*_NDM-1_, carbapenem-resistant hypervirulent *Klebsiella pneumoniae*

## Abstract

Dissemination of hypervirulent and carbapenem-resistant Klebsiella pneumoniae (CRKP) has been reported worldwide, posing a serious threat to antimicrobial therapy and public health. Outer membrane vesicles (OMVs) act as vectors for the horizontal transfer of virulence and resistance genes. However, K. pneumoniae OMVs that transfer carbapenem resistance genes into hypervirulent K. pneumoniae (hvKP) have been insufficiently investigated. Therefore, this study investigates the transmission of the *bla*_NDM-1_ gene encoding resistance via OMVs released from CRKP and the potential mechanism responsible for the carbapenem-resistant hypervirulent K. pneumoniae (CR-hvKP) emergence. OMVs were isolated via ultracentrifugation from CRKP with or without meropenem selective pressure. OMVs were then used to transform classical K. pneumoniae (ckp) ATCC 10031, extended-spectrum β-lactamase (ESBL)-producing K. pneumoniae ATCC 700603, and hvKP NTUH-K2044. Our results showed that meropenem treatment resulted in changes in the number and diameter of OMVs secreted by CRKP. OMVs derived from CRKP mediated the transfer of *bla*_NDM-1_ to ckp and hvKP, thereby increasing the carbapenem MIC of transformants. Further experiments confirmed that NTUH-K2044 transformants exhibited hypervirulence. Our study demonstrates, for the first time, that OMVs derived from CRKP can carry *bla*_NDM-1_ and deliver resistance genes to other K. pneumoniae strains, even hvKP. The transfer of carbapenem genes into hypervirulent strains may promote the emergence and dissemination of CR-hvKP. This study elucidates a new mechanism underlying the formation of CR-hvKP.

## INTRODUCTION

Klebsiella pneumoniae is a major opportunistic pathogen that causes community-acquired and hospital-acquired infections. K. pneumoniae can acquire exogenous resistance- and virulence-encoding genetic elements. Carbapenem-resistant K. pneumoniae (CRKP) has been spreading rapidly worldwide, posing a serious challenge to antimicrobial therapy (1). Hypervirulent K. pneumoniae (hvKP) can cause community-acquired infections and severe invasive infections, even in community-dwelling healthy hosts, which can result in septic liver abscesses, endophthalmitis, and meningitis (2).

The generation of K. pneumoniae strains that are hypervirulent (hv) and carbapenem resistant poses a serious threat to public health (3) and has three different evolutionary paths: hvKP strains may acquire drug-resistant plasmids, forming carbapenem-resistant hypervirulent K. pneumoniae (CR-hvKP) (4–7); CRKP strains may acquire virulence plasmids, forming hv-CRKP (8); and virulence and carbapenem resistance genetic elements may integrate into a single plasmid (9). In hvKP, horizontal gene transfer (HGT) is constrained by the substantially thickened polysaccharide capsule and the limited chromosomal recombination; the reduced plasmid diversity results in a reduction in pangenomic diversity (10). These factors mean that CRKP clones acquire virulence plasmids more efficiently than hvKP clones acquire drug-resistant plasmids. Although difficult, hvKP can acquire drug-resistant plasmids and generate CR-hvKP. Developing a better understanding of the mechanisms contributing to the transmission of drug-resistant plasmids that promote the emergence of CR-hvKP is critical.

HGT is an important mechanism leading to the transfer of drug resistance and virulence genes. Currently, the most widely described HGT mechanisms are conjugation, transformation, and transduction (11–13). Although these mechanisms conduce to gene transfer within bacteria, they have limitations, including dependence on cell-to-cell contact, bacterial competence, and host specificity. Recent studies have reported a new mechanism of outer membrane vesicles (OMVs) mediating HGT.

OMVs are 20- to 250-nm spherical bilayer liposomes, naturally released by Gram-negative bacteria during growth. OMVs originate from the outer membrane and enclose lipopolysaccharide (LPS), phospholipids, genetic elements (DNA, RNA, and plasmids), and periplasmic as well as cytoplasmic protein components (14). As part of a secretion-delivery system, OMVs allow long-distance dissemination of bacterial components to the environment and facilitate interactions between cells without the need for direct contact. The components within the lumen of vesicles are protected from DNase, making OMVs an effective HGT vector (15).

Since 1999, studies have reported that OMVs can carry virulence and resistance genes that can be horizontally transferred to bacteria (16). Dell’Annunziata et al. (17) discovered that genetically engineered KP-OMVs could transfer drug-resistant plasmids horizontally and that the transfer frequency correlated with the plasmid copy number. Subsequently, Hua et al. (10) showed that hvKP-OMVs mediated the transmission of virulence genes to extended-spectrum β-lactamase (ESBL)-producing classical K. pneumoniae (ckp). However, it is uncertain whether OMVs derived from CRKP can mediate the dissemination of drug resistance genes to hvKP, thus producing CR-hvKP. In this study, we aimed to investigate the transmission of the *bla*_NDM-1_ gene via OMVs released from CRKP and the potential mechanism responsible for the emergence of CR-hvKP.

## RESULTS

### Characterization of OMVs derived from CRKP under different conditions.

**FIG 1:**
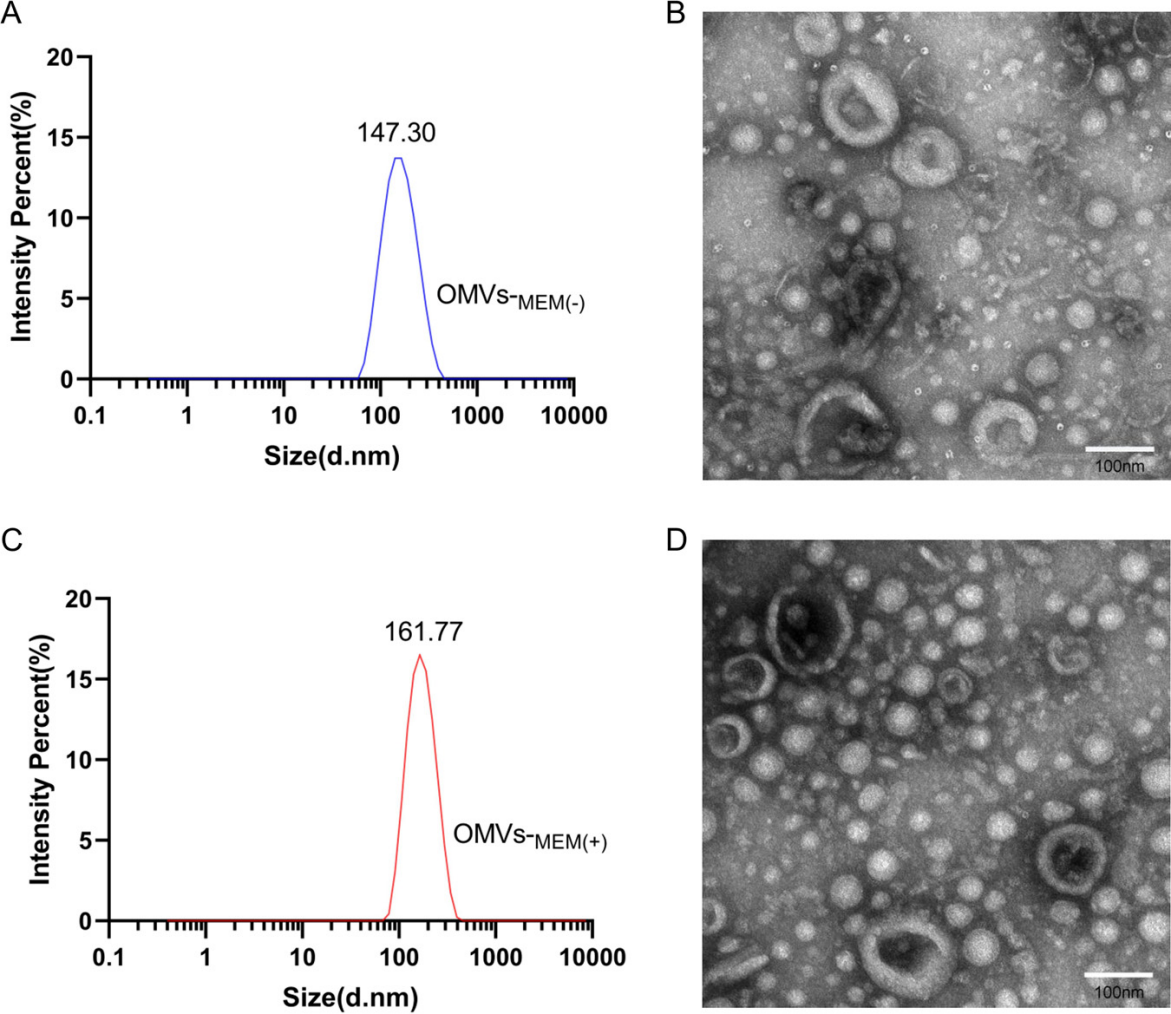
Purification and characterization of OMVs derived from CRKP under different conditions. (A and C) DLS of OMVs produced in LB broth without 8 μg/mL meropenem [OMVs-_MEM(−)_] (A) and OMVs produced in LB broth with 8 μg/mL meropenem [OMVs-_MEM(+)_] (*n* = 3) (C). (B and D) TEM of OMVs-_MEM(−)_ (B) and (D) OMVs-_MEM(+)_ (D). Bar, 100 nm.

OMVs were extracted and purified from CRKP by ultracentrifugation. Transmission electron microscopy (TEM) showed that the purified OMVs were spherical. There were no substantial morphological differences between OMVs-_MEM(−)_ (Fig. 1B) and OMVs-_MEM(+)_ (Fig. 1D). The dynamic light scattering (DLS) analysis showed that OMVs-_MEM(+)_ were larger (78.8 to 396 nm, median size of 161.77 nm) (Fig. 1C) than OMVs-_MEM(−)_ (68.1 to 396 nm, median size of 147.30 nm) (Fig. 1A). No bacteria were visualized under the microscope, and the contamination controls on the culture plates did not show any growth, thus indicating that the OMVs were well purified.

### Protein and DNA packaging in the OMVs.

**FIG 2:**
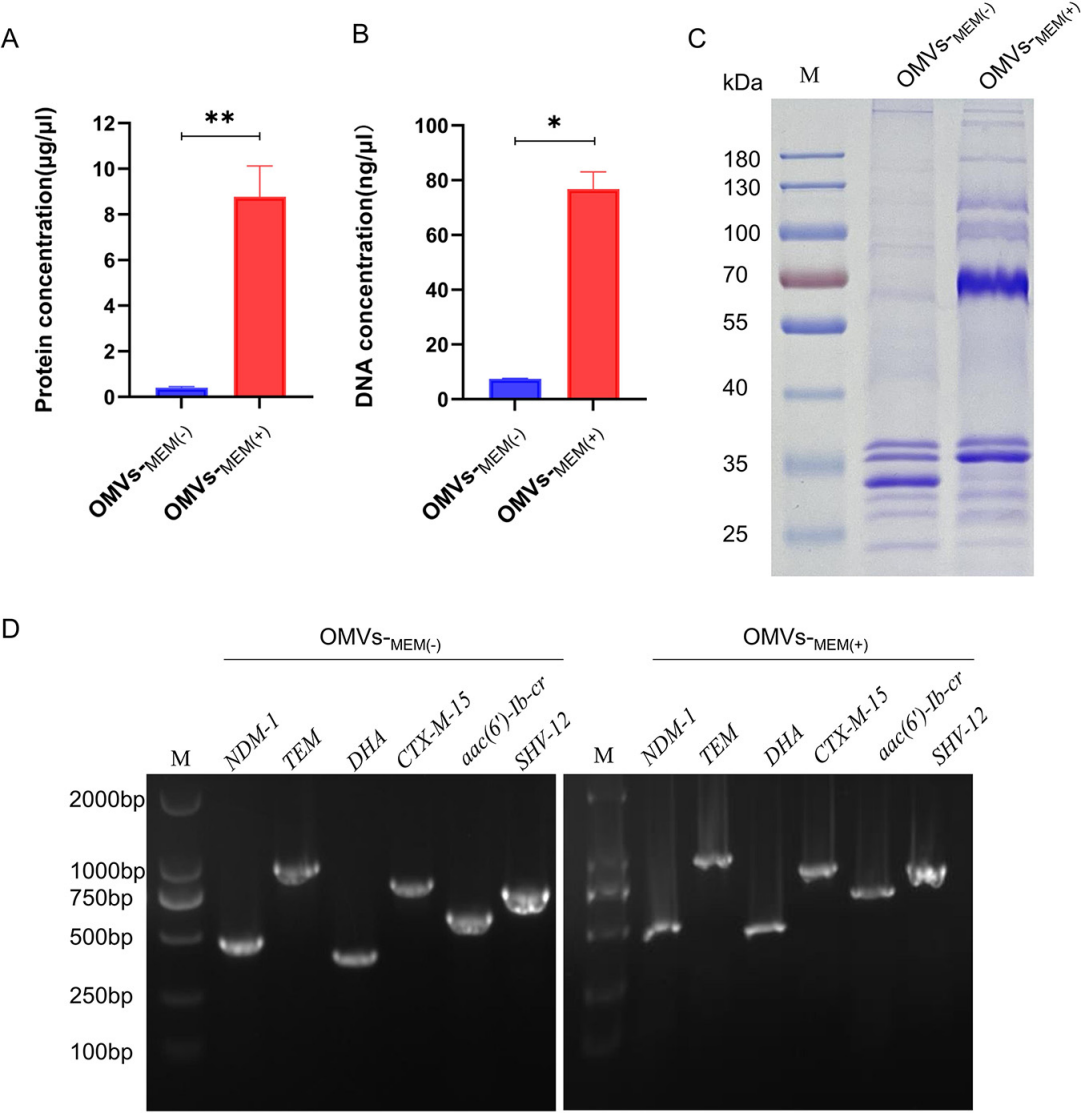
Comparative analysis of OMVs under different conditions. (A) Protein concentration of OMVs produced in LB broth without or with the pressure of 8 μg/mL meropenem [OMVs-_MEM(−)_ and OMVs-_MEM(+)_, respectively] (*n* = 3). (B) DNA concentration of OMVs-_MEM(−)_ and OMVs-_MEM(+)_ (*n* = 3). (C) Protein profiles presented using gel electrophoresis of OMVs-_MEM(−)_ and OMVs-_MEM(+)_. (D) Detection of resistance genes in OMVs-_MEM(−)_ and OMVs-_MEM(+)_ using PCR. *, *P* < 0.05, and **, *P* < 0.01.

The mean protein concentrations (based on the averages from the three experiments) of OMVs-_MEM(−)_ and OMVs-_MEM(+)_ were 0.405 ± 0.045 μg/μL and 8.777 ± 1.346 μg/μL, respectively (Fig. 2A). The mean DNA concentrations (based on the average from the three experiments) of OMVs-_MEM(−)_ and OMVs-_MEM(+)_ were 7.49 ± 0.14 ng/μL and 76.80 ± 6.21 ng/μL, respectively (Fig. 2B). The DNA purified from OMVs-_MEM(−)_ and OMVs-_MEM(+)_ gave specific amplified products for *bla*_NDM-1_, *bla*_CTX-M-15_, *bla*_SHV-12_, *bla*_TEM-1_, *bla*_DHA-1_, and *aac(6′)-Ib-cr* (Fig. 2D). SDS-PAGE was performed on purified OMVs to compare the differences in the protein composition and expression levels in OMVs-_MEM(−)_ and OMVs-_MEM(+)_. The OMVs-_MEM(−)_ and OMVs-_MEM(+)_ protein profiles showed differences in both protein composition and expression levels. Substantially elevated levels of a protein band at approximately 70 kDa were observed in OMVs-_MEM(+)_ compared with that of OMVs-_MEM(−)_ (Fig. 2C).

### OMVs from CRKP can mediate the transfer of *bla*_NDM-1_ to ckp and hvKP.

**FIG 3:**
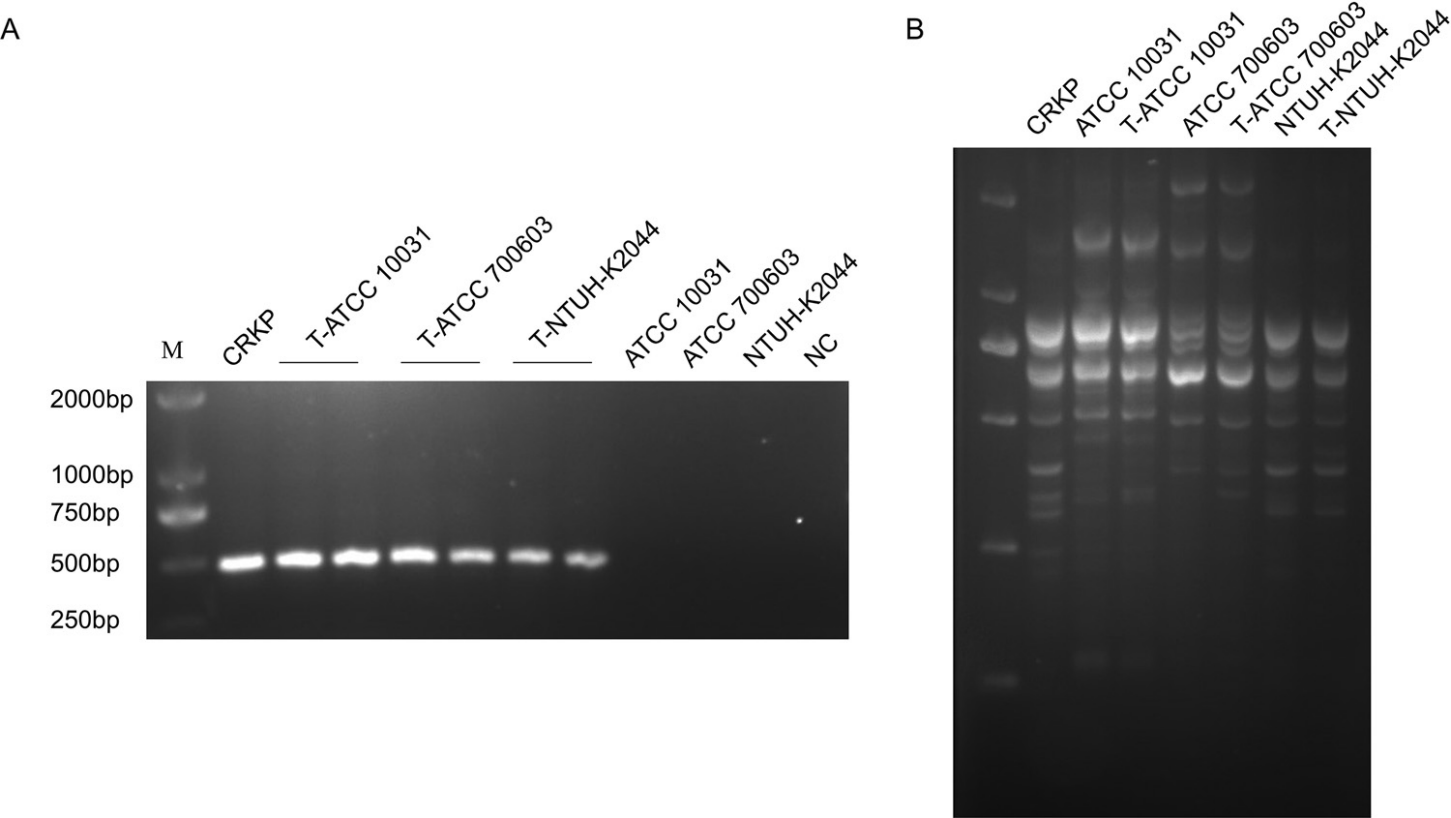
HGT via OMVs derived from CRKP. (A) Colony PCR detection of *bla*_NDM-1_ from three groups of transformants [ATCC 10031 coincubation with OMVs-_MEM(+)_, ATCC 700603 coincubation with OMVs-_MEM(+)_, and NTUH-K2044 coincubation with OMVs-_MEM(+)_]. Two colonies were randomly selected from each of the three groups for the detection of *bla*_NDM-1_. Genomic DNA of CRKP was used as the positive control, and genomic DNA of ATCC 10031, ATCC 700603, and NTUH-K2044 and sterile water were used as the negative controls (NC). (B) REP-PCR profile of the donor, recipient, and transformant strains used in this study. T, transformant.

ckp ATCC 10031, ckp ATCC 700603, and hvKP NTUH-K2044 were used as recipient strains and were coincubated with OMVs isolated from CRKP. DNA transformation was first ruled out because no colonies grew on the MacConkey (MAC) plate with 1 μg/mL meropenem when the plasmid was incubated with ckp and hvKP. Additionally, the group of lysed OMVs showed no colonies on the MAC plate with 1 μg/mL meropenem, and only intact OMVs led to meropenem-resistant colony growth. Thus, we concluded that only intact OMVs could mediate the transfer of *bla*_NDM-1_. The *bla*_NDM-1_ transfer frequencies from 500 μg OMVs-_MEM(+)_ to ckp ATCC 10031 and hvKP NTUH-K2044 were 9.79 × 10^−9^ ± 3.52 × 10^−9^ and 7.83 × 10^−9^ ± 3.48 × 10^−9^, respectively. However, the transfer frequency from 200 μg OMVs-_MEM(+)_ to ATCC 700603 was much higher (8.08 × 10^−7^ ± 1.89 × 10^−7^). When ATCC 700603 was coincubated with 20 μg OMVs, the transfer frequency of the OMVs-_MEM(−)_ group (1.42 × 10^−6^ ± 0.27 × 10^−6^) was higher than that of the OMVs-_MEM(+)_ group (1.65 × 10^−7^ ± 0.25 × 10^−7^) (Table 1). Two randomly selected transformants from each of the three recipient strain groups were confirmed for *bla*_NDM-1_ using PCR and were visualized using agarose gel electrophoresis. All strains were *bla*_NDM-1_-positive strains (Fig. 3A). However, all strains were negative for *bla*_CTX-M-15_, *bla*_SHV-12_, *bla*_TEM-1C_, *bla*_DHA-1_, and *aac(6′)-Ib-cr*. To completely understand the results, the whole genome of the NTUH-K2044 transformants was sequenced (GenBank accession number SAMN33420516). The results showed that the plasmid IncFIB_pKPHS1_ harboring *bla*_NDM-1_ was transferred to NTUH-K2044 via OMVs, and *bla*_NDM-1_ was the only resistance gene transferred.

**TABLE 1:** Frequencies of *bla*_NDM-1_ transfer via OMVs from CRKP into recipient bacteria

Treatment	Amt of OMVs (μg)	Transfer frequency
ATCC 10031 + OMVs-_MEM(+)_	500	(9.79 ± 3.52) × 10^−9^
NTUH-K2044 + OMVs-_MEM(+)_	500	(7.83 ± 3.48) × 10^−9^
ATCC 700603 + OMVs-_MEM(+)_	200	(8.08 ± 1.89) × 10^−7^
ATCC 700603 + OMVs-_MEM(+)_	20	(1.65 ± 0.25) × 10^−7^
ATCC 700603 + OMVs-_MEM(−)_	20	(1.42 ± 0.27) × 10^−6^

Repetitive extragenic palindromic PCR (REP-PCR) was performed on the donors, recipients, and transformants. There was no difference in the REP-PCR amplification profiles between recipient strains and transformants (Fig. 3B). Therefore, contamination by the donor strain was excluded.

Antibiotic sensitivity tests were performed on donors, recipients, and transformants. The CRKP strain showed high β-lactam resistance, including cephalosporin and carbapenem resistance. The transformants showed higher MIC values for β-lactam antibiotics than their respective recipient strains (Table 2). Moreover, there was no difference in the MIC values of ciprofloxacin, indicating that the transformants did not acquire *aac(6′)-Ib-cr*.

**TABLE 2:** Antibiotic susceptibility profiles of donor, recipient and transformant strains

Antibiotic^*a*^	MIC (μg/mL) for strain:						
	CRKP	ATCC 10031^*c*^	T-ATCC 10031^*b*^	ATCC 700603^*d*^	T-ATCC 700603	NTUH-K2044^*e*^	T-NTUH-K2044
AMP	≥32	16	≥32	≥32	≥32	≥32	≥32
AMC	≥32	≤2	≥32	4	≥32	≤2	≥32
TZP	≥128	≤4	64	8	64	≤4	64
CFZ	≥64	≤4	≥64	≤4	≥64	≤4	≥64
FOX	≥64	≤4	≥64	≥64	≥64	≤4	≥64
CRO	≥64	≤1	≥64	≤1	≥64	≤1	≥64
FEP	≥64	≤1	2	≤1	8	≤1	2
ATM	≥64	≤1	≤1	≤1	≤1	≤1	≤1
MEM	16	≤0.5	4	≤0.5	4	≤0.5	4
ETP	≥8	≤0.5	4	≤0.5	≥8	≤0.5	4
IPM	4	≤1	4	≤1	4	≤1	4
AMK	≤2	≤2	≤2	≤2	≤2	≤2	≤2
GEN	≤1	≤1	≤1	≤1	≤1	≤1	≤1
TOB	8	≤1	≤1	≤1	≤1	≤1	≤1
CIP	1	≤0.25	≤0.25	≤0.25	≤0.25	≤0.25	≤0.25
LVX	1	≤0.25	≤0.25	1	1	≤0.25	≤0.25
TGC	2	≤0.5	≤0.5	≥8	≥8	≤0.5	≤0.5
NIT	64	≤16	≤16	64	64	64	64
SXT	≥320	≤20	≤20	≤20	≤20	≤20	≤20

a AMP, ampicillin; AMC, amoxicillin-clavulanic acid; TZP, piperacillin-tazobactam; CFZ, cefazolin; FOX, cefoxitin; CRO, ceftriaxone; FEP, cefepime; ATM, aztreonam; MEM, meropenem; ETP, ertapenem; IPM, imipenem; AMK, amikacin; GEN, gentamicin; TOB, tobramycin; CIP, ciprofloxacin; LVX, levofloxacin; TGC, tigecycline; NIT, nitrofurantoin; SXT, trimethoprim-sulfamethoxazole.

b T, transformant.

c ATCC 10031, K. pneumoniae reference strains.

d ATCC 700603 produces β-lactamase SHV-18 and is a CLSI quality control strain for antimicrobial susceptibility testing.

e NTUH-K2044, isolated from a Taiwanese liver abscess, is widely used as a model strain for hvKP.

### Virulence level of NTUH-K2044 transformants.

**FIG 4:**
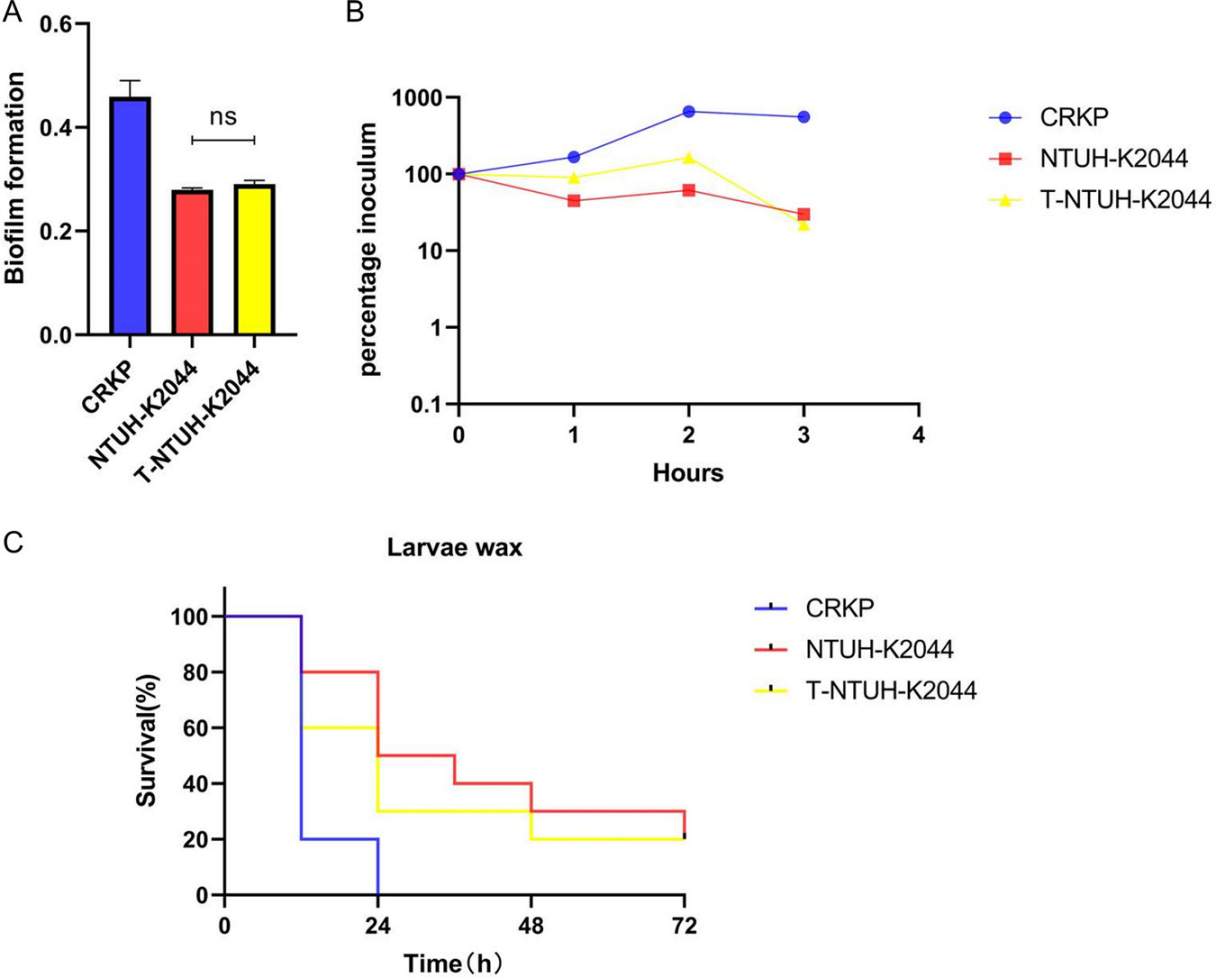
Virulence phenotype of donor, recipient, and transformant strains. (A and B) Biofilm formation (A) and serum resistance (B) of CRKP, NTUH-K2044, and NTUH-K2044 transformants. (C) Virulence level of different strains as depicted in a wax moth larva infection model. ns, *P* > 0.05. T, transformant.

Compared to NTUH-K2044, NTUH-K2044 transformants harboring *bla*_NDM-1_ showed no significant difference in biofilm formation (*P* > 0.05) or serum resistance (Fig. 4A and B), and high virulence was supported by larva infection experiments (Fig. 4C). These data confirmed that the virulence of transformants that acquired drug-resistant genes was not reduced compared to that of NTUH-K2044 recipient bacteria.

**FIG 5:**
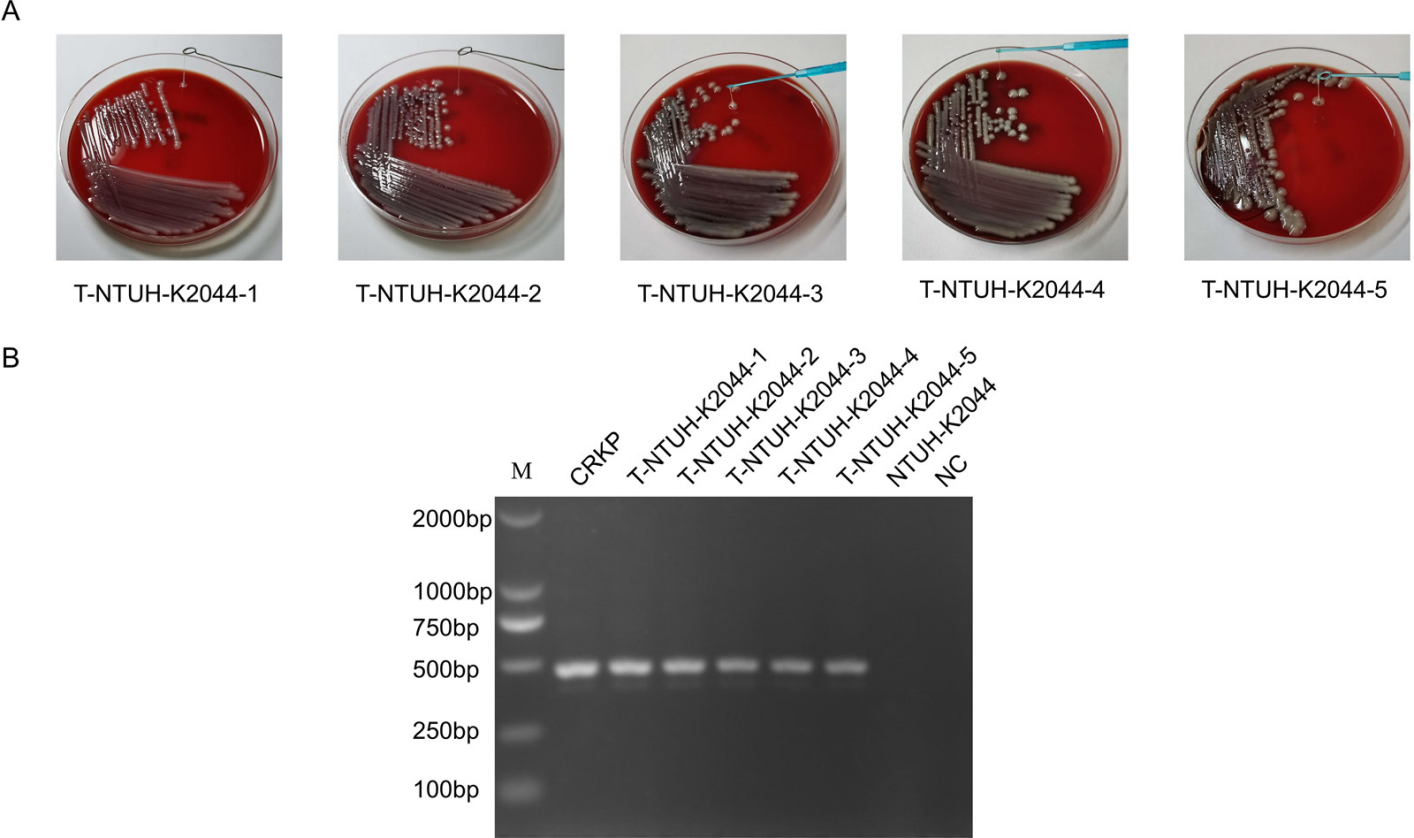
NTUH-K2044 transformants exhibited a dual phenotype of hypermucoviscosity and carbapenem resistance after several generations. (A) String tests on blood agar plates of five generations of NTUH-K2044 transformants. (B) Colony PCR detection of *bla*_NDM-1_ of five generations of NTUH-K2044 transformants. T, transformant.

To confirm whether the NTUH-K2044 transformants exhibited a stable hypermucoviscosity phenotype, NTUH-K2044 transformants were passaged for five generations and the hypermucoviscosity phenotype of each of these generations was measured. String tests on blood agar plates showed that *bla*_NDM-1_-positive NTUH-K2044 still exhibited a hypermucoviscosity phenotype (Fig. 5A). The *bla*_NDM-1_ of colonies from these generations was analyzed using PCR, which showed that the transformants that acquired the resistance genes still maintained *bla*_NDM-1_ after several generations (Fig. 5B).

### OMVs from CRKP can carry NDM-1 and hydrolyze meropenem.

**FIG 6:**
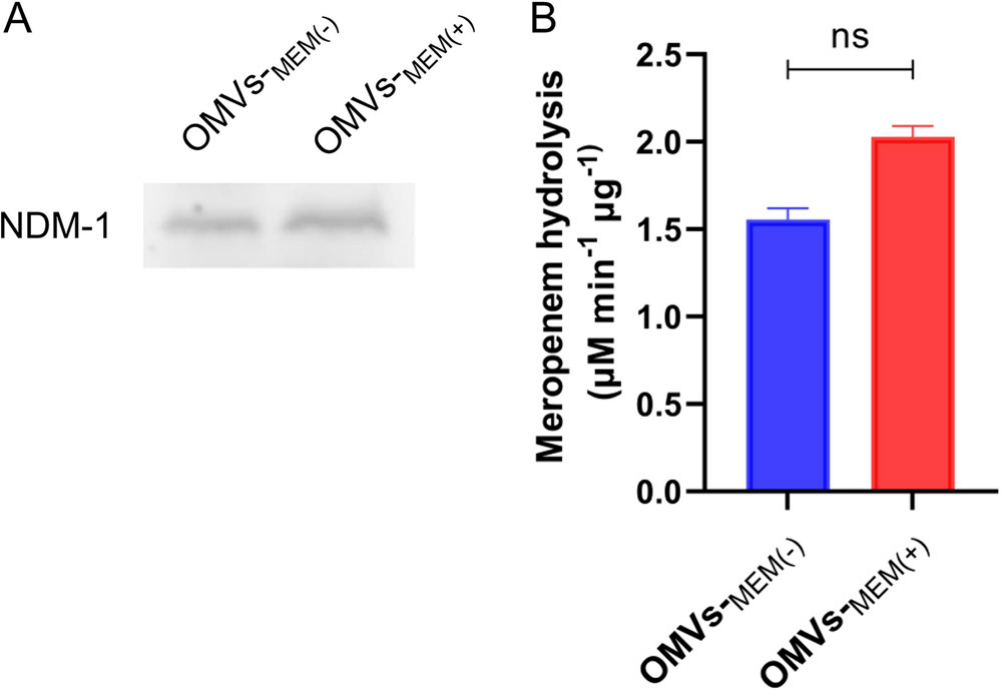
(A) Immunodetection of NDM-1 in OMVs produced in LB broth without or with 8 μg/mL meropenem [OMVs-_MEM(−)_ and OMVs-_MEM(+)_, respectively] using Western blotting. (B) Meropenem hydrolysis by OMVs-_MEM(−)_ and OMVs-_MEM(+)_ (*n* = 3). ns, *P* > 0.05.

To verify whether OMVs isolated from CRKP carry metallo-β-lactamases, Western blot analysis was performed using rabbit anti-NDM-1 antibodies. Results showed that OMVs-_MEM(−)_ and OMVs-_MEM(+)_ contained NDM-1 (Fig. 6A). To determine the NDM-1 activity, meropenem was used as the substrate, and the hydrolysis rates of OMVs-_MEM(−)_ and OMVs-_MEM(+)_ were 1.556 ± 0.052 μM min^−1^ μg^−1^ and 2.028 ± 0.052 μM min^−1^ μg^−1^, respectively (Fig. 6B).

## DISCUSSION

The World Health Organization (WHO) antimicrobial resistance surveillance report addressed the severity of CRKP, which has a prevalence of >50% in some regions (18). In China, NDM carbapenemase is the second most prevalent carbapenemase after KPC-2 (19). The exchange of genes is common between bacterial species, leading to the rapid spread of resistance. Some features of hvKP make it difficult to acquire genetic material from the environment, including genetic restrictions and the constraints of the significantly thickened capsule. Nevertheless, carbapenem resistance genes have gradually spread to hvKP, leading to the emergence of CR-hvKP (5–7); this represents a significant challenge for antimicrobial therapy. An improved understanding of the mechanisms underlying the spread of resistance genes is needed to limit this serious threat to global health.

In addition to the three classical mechanisms of HGT (conjunction, transformation, and transduction), OMVs are a recently proposed mechanism that might allow HGT. Studies have reported that OMVs carrying resistance and virulence genes have the potential to be delivered to other bacteria (16, 17, 20, 21, 22). Moreover, a recent study has demonstrated that K. pneumoniae can transfer virulence genes to carbapenem-resistant bacteria via OMVs (23). However, OMVs derived from K. pneumoniae that transfer carbapenem resistance genes to hypervirulent K. pneumoniae have been insufficiently investigated.

In this study, OMVs were isolated from CRKP without or with 8-μg/mL meropenem treatment [OMVs-_MEM(−)_ and OMVs-_MEM(+)_, respectively]. The TEM and DLS results showed that both OMVs-_MEM(−)_ and OMVs-_MEM(+)_ were spherical in structure and had no substantial differences in morphology. However, the mean particle size of OMVs-_MEM(+)_ (161.77 nm) was larger than that of OMVs-_MEM(−)_ (147.30 nm), and the protein as well as DNA content of OMVs-_MEM(+)_ was also higher. These differences can be attributed to the antibiotic selection pressure. According to a previous study (24), imipenem enhances the release of OMVs from multidrug-resistant K. pneumoniae, indicating that the generation of OMVs depends on environmental factors. In this study, the protein profiles showed that OMVs-_MEM(+)_ had a protein band at approximately 70 kDa which was substantially elevated compared to that of OMVs-_MEM(−)_ (Fig. 2C). This is in line with the previously presented results (24). The composition and function of the 70-kDa protein identified in the present study require further investigation.

Our experimental results showed that OMVs derived from CRKP could mediate the transfer of *bla*_NDM-1_ to ckp, generating CRKP and therefore increasing the carbapenem MIC of the transformants. This demonstrated that, in addition to *bla*_KPC-2_ (23), *bla*_NDM-1_ could also be transferred between K. pneumoniae cells via OMVs. Another important and novel finding from our study is the transfer of *bla*_NDM-1_ to hvKP, indicating the potential for the emergence of CR-hvKP via OMV-mediated HGT. The frequency of OMV-mediated *bla*_NDM-1_ transfer to hvKP was slightly lower than that to ckp, suggesting that the gene transfer frequency may be related to the characteristics of the recipient strains. Further exploration is needed regarding the factors affecting the frequency of OMV-mediated transfer of drug-resistant plasmids, like the nature of the capsule polysaccharide. Although more OMVs were formed after meropenem treatment, the frequency of *bla*_NDM-1_ gene transfer was not increased compared to that in the untreated group, indicating that the OMVs produced by CRKP following meropenem treatment may have additional, unidentified roles. In addition, despite the inclusion of multiple drug resistance genes in the OMVs, we found that only *bla*_NDM-1_ was transferred to the recipient strains, suggesting a selective mechanism that remains to be further investigated.

A previous study (25) reported that the CR-hvKP strain formed by conjugative transfer of the KPC plasmid to NTUH-K2044 presented as nonmucoid colonies in the third generation. Compared with the transconjugant mucoid strain, the nonmucoid strain showed a much higher level of resistance to carbapenems but exhibited significantly decreased virulence levels. However, the CR-hvKP strain formed in our study retained the hypermucoviscosity phenotype throughout the five passages (Fig. 5A). The NTUH-K2044 transformants showed no decrease in biofilm formation and serum resistance, and larva infection experiments showed that these strains retained high virulence. Therefore, these results demonstrated that the CR-hvKP strain formed by OMV-mediated transfer of *bla*_NDM-1_ had the potential for a dual phenotype of carbapenem resistance and hypervirulence without significant burdens.

Previous studies have revealed that OMVs secreted from antibiotic-resistant bacteria act as efficient and protective β-lactamase and NDM-1 transporters and can contribute to antibiotic resistance in a nongenetic manner in the bacterial community (26, 27). Our results showed that OMVs derived from CRKP can carry NDM-1 and hydrolyze meropenem. The simultaneous secretion of proteins and genetic elements suggests that OMVs contribute to the spread of antimicrobial resistance, not only by the inactivation of antibiotics through enzyme transport but also by transferring genetic elements to antibiotic-sensitive bacteria. This indicates that new therapeutic options that interfere with vesicle secretion should be explored.

The main limitation of this study is that the mechanism of DNA wrapping into OMVs and the mechanism by which vesicles enter recipient bacterial strains remain to be elucidated. Our future studies will aim to study the selective mechanism underlying transfer of *bla*_NDM-1_ and shed light on whether CR-hvKP has the potential to continuously disseminate drug resistance genes.

In summary, our study demonstrated for the first time that OMVs derived from CRKP could carry *bla*_NDM-1_ and deliver resistance genes to other K. pneumoniae strains, even hvKP. The transfer of carbapenem resistance genes into hypervirulent strains may promote the emergence and dissemination of CR-hvKP. This study elucidates a new mechanism of HGT which increases the risk of generating CR-hvKP, a concerning development in the fight against antimicrobial resistance.

## MATERIALS AND METHODS

### Bacterial strains.

CRKP carrying the *bla*_NDM-1_ gene was used for the isolation and purification of OMVs. This clinical strain was isolated from the blood of a pediatric patient in 2018 from a tertiary hospital. This strain is reported to harbor *bla*_NDM-1_, *bla*_CTX-M-15_, *bla*_SHV-12_, *bla*_TEM-1C_, *bla*_DHA-1_, and *aac(6′)-Ib-cr* resistance determinants based on the whole-genome sequence (28). The CRKP genome contains a chromosome of 5,344,963 bp and four plasmids, pCRKP-1 to -4, with sizes of 262,673 bp, 162,879 bp, 124,316 bp, and 3,681 bp, respectively. Plasmid pCRKP-2 belongs to the IncFIB_pKPHS1_/IncX3 replicon and harbors β-lactam resistance genes *bla*_NDM-1_ and *bla*_SHV-12_; *bla*_SHV-12_ is not in the neighboring region with *bla*_NDM-1_. Plasmid pCRKP-1 contains *bla*_DHA-1_, while pCRKP-3 contains *bla*_CTX-M-15_, *bla*_TEM-1C_, and *aac(6′)-Ib-cr*. The genome sequence of CRKP was deposited in GenBank (accession number SAMN33420447).

K. pneumoniae ATCC 10031, ESBL-producing K. pneumoniae ATCC 700603, and hvKP NTUH-K2044 were used as recipient strains for transformation experiments.

### OMV purification.

CRKP was incubated in Luria-Bertani (LB) medium (Sangon Biotech, China) without antibiotics or with 8 μg/mL meropenem at 37°C and 200 rpm for 12 h and then used for the preparations of OMVs-_MEM(−)_ and OMVs-_MEM(+)_, respectively. Cell cultures were centrifuged at 4°C and 3,000 × *g* for 15 min, and supernatants were filtered through a 0.22-μm membrane (Merck Millipore, Germany) to remove small cell debris. The filtrates were concentrated using a 100-kDa 50-mL ultrafiltration tube (Millipore) and then subjected to ultracentrifugation at 4°C and 100,000 × *g* for 3 h using a 70 Ti rotor (Beckman, USA). The pellets were washed with sterile phosphate-buffered saline (PBS) (pH 7.4). Ultracentrifugation was repeated to obtain OMVs. The pellet containing OMVs was resuspended in 200 μL of PBS, and OMVs were cultured on blood agar plates to ensure sterility. The OMVs were stored at −80°C until used.

### Quantification of OMV proteins and DNA in OMVs.

OMV protein concentrations were determined using a bicinchoninic acid (BCA) protein assay kit (Beyotime Biotechnology, China), according to the manufacturer’s protocol. Intravesicular DNA was quantified using a previously described method (20) with a few modifications. Briefly, 10 μg of OMVs was treated with 1 U DNase I (Thermo Scientific, USA) and 100 μg/mL proteinase K (Tiangen Biotech, China). DNase I- and proteinase K-treated vesicles were subsequently lysed with 0.125% Triton X-100 (Solarbio, China) solution for 30 min at 37°C, before the DNA was recovered using a DNA purification kit (Vazyme, China), according to the manufacturer’s protocol. The DNA concentration and purity were determined using a NanoDrop spectrophotometer (Thermo Scientific).

### TEM.

Purified OMVs were visualized using a transmission electron microscope (TEM; TEM-1400Plus; Japan) with negative staining. OMVs (30 μL) were absorbed onto a carbon-coated Formvar film and negatively stained with 2.0% (wt/vol) uranyl acetate at room temperature for 5 min. Samples were observed under a TEM operating at 120 kV.

### DLS.

The particle size and polydispersity index of the OMVs were determined using a Zeta-sizer nano-ZS (Malvern Instruments, UK). OMV samples were diluted to a concentration of 20 μg/mL with sterile PBS buffer, and 1 mL of these OMV aliquots was pipetted into a sterile cuvette. All measurements were performed at 25°C, and experiments were performed in triplicate.

### PCR.

The presence of resistance genes [*bla*_NDM-1_, *bla*_CTX-M-15_, *bla*_SHV-12_, *bla*_TEM-1C_, *bla*_DHA-1_, and *aac(6′)-Ib-cr*] was detected using PCR. The genomic DNA of K. pneumoniae was extracted using the boiling method. The PCR products were visualized on a 1% agarose gel. Primers used in this study are listed in Table 3.

**TABLE 3:** Oligonucleotides used in this study

Primer	Sequence (5′–3′)	Length (bp)	Use
*NDM-1* Forw	GGGCAGTCGCTTCCAACGGT	476	PCR
*NDM-1* Rev	GTAGTGCTCAGTGTCGGCAT		
*CTX-M-15* Forw	TTGTTAGGAAGTGTGCCGCT	819	PCR
*CTX-M-15* Rev	TTACAAACCGTCGGTGACGA		
*SHV-12* Forw	TCTCCCTGTTAGCCACCCT	793	PCR
*SHV-12* Rev	ATTTGCTGATTTCGCTCGGC		
*TEM* Forw	GTGCGCGGAACCCCTATT	919	PCR
*TEM* Rev	TTACCAATGCTTAATCAGTGAGGC		
*DHA* Forw	AACTTTCACAGGTGTGCTGGGT	405	PCR
*DHA* Rev	CCGTACGCATACTGGCTTTGC		
*aac(6′)-Ib-cr* Forw	ATATGCGGATCCAATGAGCAACGCAAAAACAAAGTTAG	544	PCR
*aac(6′)-Ib-cr* Rev	ATAGCGAATTCTTAGGCATCACTGCGTGTTCGCTC		
REP Forw	IIIGCGCCGICATCAGGC	–^*a*^	REP-PCR
REP Rev	ACGTCTTATCAGGCCTAC		

a no specific length.

### OMV-mediated *bla*_NDM-1_ transfer.

OMV-mediated *bla*_NDM-1_ transfer experiments were performed as previously described (21), with slight modifications. ckp ATCC 10031, ckp ATCC 700603, and hvKP NTUH-K2044 (recipient strains) were grown in LB broth at 37°C and 200 rpm for 4 to 5 h to an optical density at 600 nm (OD_600_) of 0.4. Cells were centrifuged and resuspended in cold LB broth to a final concentration of 10^7^ CFU/mL. These suspensions (100 μL) were mixed with OMV-_MEM(−)_ or OMV-_MEM(+)_ and incubated statically at 37°C for 4 h, followed by another 4 h of shaking (180 rpm). LB broth was added to a final volume of 10 mL, and the incubation was continued overnight at 37°C with shaking (180 rpm). Bacterial cultures were centrifuged and resuspended in 1 mL of fresh LB broth. A 100-μL aliquot of the suspension was incubated overnight on MAC agar (for total bacterial counts) or MAC agar supplemented with 1 μg/mL meropenem (for transformant selection). The frequency of OMV-mediated *bla*_NDM-1_ transfer was calculated as the number of transformants (CFU per milliliter) divided by the total bacterial count (CFU per milliliter). Two randomly selected transformants from each recipient were confirmed to harbor *bla*_NDM-1_ by PCR.

To demonstrate that DNA transfer was mediated by OMVs, two sets of experiments were performed: (i) plasmid DNA isolated from CRKP and (ii) OMVs lysed using 0.125% Triton X-100. All experiments were performed in duplicate.

### REP-PCR.

Repetitive extragenic palindromic PCR (REP-PCR) was performed as previously described (29). The primers used to perform REP-PCR are listed in Table 3. Two strains were considered to belong to the same genotype if they had the same pattern of bands, and a maximum difference of two band patterns was considered acceptable.

### Antibiotic susceptibility testing.

Antibiotic susceptibility testing of the donor, recipient, and transformant strains was performed using broth microdilution. The MICs of ampicillin, amoxicillin-clavulanic acid, piperacillin-tazobactam, cefazolin, cefoxitin, ceftriaxone, cefepime, aztreonam, meropenem, ertapenem, imipenem, amikacin, gentamicin, tobramycin, ciprofloxacin, levofloxacin, tigecycline, nitrofurantoin, and trimethoprim-sulfamethoxazole were determined according to the Clinical and Laboratory Standards Institute (30).

### Biofilm formation assay.

Biofilm formation assays were implemented as previously described (31), with slight modifications. In brief, 200 μL of bacterial culture (1.5 × 10^6^ CFU/mL) was added to 96-well flat-bottomed plates and incubated for 24 h at 37°C. Cultures were subsequently removed and washed three times with PBS. The wells were dried, stained with 1% crystal violet for 15 min, and then washed three times with distilled water. The absorbance was measured at 570 nm after solubilization with 95% ethanol. Each assay was performed in duplicate and independently repeated three times.

### Serum resistance assay.

The serum resistance experiments were performed as previously described (31). Bacteria grown to the mid-log phase were mixed with normal human serum in a 1:3 ratio and then incubated at 37°C for 3 h. At 1-h intervals following incubation, serially diluted bacteria were plated on Mueller-Hinton agar. Serum resistance was determined by plotting the survival percentage of each strain against the incubation time. All experiments were performed in triplicate.

### Larva infection models.

Galleria mellonella larvae weighing 200 to 400 mg, purchased from Tianjin Huiyude Biotechnology Company, were used to determine the virulence levels of donor, recipient, and transformant strains. The concentration of the strain was adjusted to 1 × 10^8^ CFU/mL. Each group of 10 G. mellonella larvae was injected with 10 μL of bacterial culture (10^6^ CFU) and incubated at 37°C for 72 h, and the survival rates of the larvae were observed every 12 h. Animal experiments were performed in duplicate.

### String test.

The NTUH-K2044 transformant strain was incubated on a blood agar plate at 37°C overnight. A positive string test was performed by stretching a viscous string with an inoculation loop; viscous strings with a length of >5 mm indicate a hypermucoviscosity phenotype.

### SDS-PAGE, NDM-1 detection, and β-lactamase activity.

Sixteen-microgram OMV samples were mixed with 5× loading buffer, denatured by heating at 100°C for 10 min, and loaded onto 10% SDS-PAGE gels. The gels were stained with Coomassie brilliant blue (Sangon Biotech, China), or the separated proteins were transferred onto the polyvinylidene difluoride (PVDF) membrane. Western blotting using the polyclonal anti-NDM-1 antibody at a 1:1,000 dilution (Novus Biologicals) and goat anti-rabbit IgG horseradish peroxidase (HRP)-conjugated secondary antibody was performed as previously described (32, 33), with slight modifications. Chemiluminescent signals were acquired using an Image Quant 800 (GE Healthcare).

The metallo-β-lactamase activity was determined by monitoring the absorbance using 256 μg/mL meropenem as the substrate in a reaction buffer containing 10 mM HEPES and 200 mM NaCl (pH 7.4). One microgram of OMVs was added into reaction mixtures, for a total volume of 100 μL, and the absorbance at 300 nm was measured immediately in kinetic mode for 10 min. A standard curve was generated using 0 to 256 μg/mL meropenem. The NDM-1 activity of the sample was expressed as the number of micromoles of meropenem hydrolyzed per minute per microgram of protein.

### Ethics statement.

Isolates were collected from routine diagnoses, and only pure cultures of bacterial isolates were investigated. No patient-related data were analyzed. According to the regulations of Xiangya Hospital of Central South University, such research does not require ethical approval.

### Data availability.

The sequence of the whole genome of the NTUH-K2044 transformants was deposited in GenBank under accession number SAMN33420516. The genome sequence of CRKP was deposited in GenBank under accession number SAMN33420447.
